# An Axis between the Long Non-Coding RNA *HOXA11-AS* and NQOs Enhances Metastatic Ability in Oral Squamous Cell Carcinoma

**DOI:** 10.3390/ijms231810704

**Published:** 2022-09-14

**Authors:** Chie Nakashima, Rina Fujiwara-Tani, Shiori Mori, Shingo Kishi, Hitoshi Ohmori, Kiyomu Fujii, Takuya Mori, Yoshihiro Miyagawa, Kazuhiko Yamamoto, Tadaaki Kirita, Yi Luo, Hiroki Kuniyasu

**Affiliations:** 1 Department of Molecular Pathology, School of Medicine, Nara Medical University, Kashihara 634-8521, Japan; 2 Department of Oral and Maxillofacial Surgery, School of Medicine, Nara Medical University, Kashihara 634-8522, Japan; 3 Jiangsu Province Key Laboratory of Neuroregeneration, Nantong University, 19 Qixiu Road, Nantong 226001, China

**Keywords:** long non-coding RNA, microRNA, HOXA11-AS, mir-494, NQO

## Abstract

Long non-coding RNAs (lncRNAs) play critical roles in human cancers. HOXA11 anti-sense RNA (*HOXA11-AS*) is an lncRNA belonging to the homeobox (HOX) gene cluster that promotes liver metastasis in human colon cancer. However, its role and mechanism of action in human oral squamous cell carcinoma (OSCC) are unclear. In this study, we investigated *HOXA11-AS* expression and function in human OSCC tissues and cell lines, as well as a mouse model of OSCC. Our analyses showed that *HOXA11-AS* expression in human OSCC cases correlates with lymph node metastasis, nicotinamide adenine dinucleotide (NAD)(P)H: quinone oxidoreductase 1 (*NQO1*) upregulation, and dihydronicotinamide riboside (NRH): quinone oxidoreductase 2 (*NQO2*) downregulation. Using the human OSCC cell lines HSC3 and HSC4, we demonstrate that *HOXA11-AS* promotes *NQO1* expression by sponging microRNA-494. In contrast, *HOXA11-AS* recruits zeste homolog 2 (EZH2) to the *NQO2* promoter to suppress its expression via the trimethylation of H3K27. The upregulation of NQO1 enzymatic activity by *HOXA11-AS* results in the consumption of flavin adenine dinucleotide (FAD), which reduces FAD-requiring glyceraldehyde-3-phosphate dehydrogenase (GAPDH) activity and suppresses glycolysis. However, our analyses show that lactic acid fermentation levels are preserved by glutaminolysis due to increased malic enzyme-1 expression, promoting enhanced proliferation, invasion, survival, and drug resistance. In contrast, suppression of *NQO2* expression reduces the consumption of NRH via NQO2 enzymatic activity and increases NAD levels, which promotes enhanced stemness and metastatic potential. In mouse tumor models, knockdown of HOXA11-AS markedly suppressed tumor growth and lung metastasis. From these findings, targeting *HOXA11-AS* may strongly suppress high-grade OSCC by regulating both *NQO1* and *NQO2*.

## 1. Introduction

The worldwide incidence of oral squamous cell carcinoma (OSCC) is 6.2% and 3.6% in men and women, respectively, with corresponding mortality rates of 3.3% and 1.6% [1]. The frequency of this disease continues to increase [2], which is cause for concern as the 5-year survival rate for OSCC is approximately 60%, and the prognosis is poor in the advanced stages [3]. In recent years, drug therapy for head and neck squamous cell carcinoma has progressed rapidly. OSCC is typically treated with cytotoxic anticancer drugs, such as those based on platinum (cisplatin, carboplatin) and taxane (docetaxel, paclitaxel), but immune checkpoint inhibitors, such as epidermal growth factor receptor tyrosine kinase inhibitors (cetuximab), and anti-programed cell death-1 antibodies (nivolumab and pembrolizumab), are also available [4]. However, the prognosis for high-grade OSCCs remains poor. Anticancer drugs offer clear benefits for the treatment of this cancer, which makes the identification of new molecular therapeutic targets for OSCC an urgent issue.

Homeobox (HOX) genes play an important role in embryonic development and carcinogenesis. There are 39 HOX genes in four clusters in humans, and each cluster also contains numerous non-coding RNAs that do not code for proteins, including several long non-coding RNAs (lncRNAs), such as *HOXA11-AS* anti-sense RNA (*HOXA11-AS*), that are dysregulated in cancer [5]. The 1628 bp long *HOXA11-AS* gene produces an lncRNA that is involved in the growth and metastasis of malignant tumors [6], and has been reported to promote colon cancer liver metastasis [7]. *HOXA11-AS* functions as a protein scaffold for polycomb repressive complex-2, lysine-specific histone demethylase-1, and DNA methyltransferase-1, forming an RNA–protein complex that epigenetically modifies chromosomes in the nucleus. *HOXA11-AS* also sponges miRNAs as a competing endogenous RNA [8]. Furthermore, HOXA11-AS epigenetically regulates genetic expression through its action with EZH2. [9].

The quinone oxidoreductases nicotinamide adenine dinucleotide (NAD)(P)H: quinone oxidoreductase 1 (NQO1) and dihydronicotinamide riboside (NRH): quinone oxidoreductase 2 (NQO2) are flavoproteins. NQO1 catalyzes metabolic detoxification and protects cells from redox cycling and oxidative stress, whereas the function of NQO2 is unclear [10]. Their cofactor and substrate affinities are also different. NQO1 requires NAD(P)H as an electron donor, whereas NQO2 requires NRH. *NQO1* is overexpressed in many cancers and is involved in carcinogenesis, drug resistance, and cancer progression [11,12]. In contrast, decreased expression of *NQO2* correlates with liver metastasis in colorectal cancer [7]. *HOXA11-AS* is involved in NQO2 downregulation to enhance metastasis [7], but the mechanism by which it regulates the expression of NQO2 is unclear. Both NQO1 and NQO2 are oxidative stress-related genes; HOXA11-AS has been reported to repress NQO2 expression, whereas the role of HOXA11-AS in NQO1 expression is unclear. Furthermore, the roles of *HOXA11-AS* and NQOs in OSCC remain poorly understood.

In this study, we elucidated the roles and interactions of *HOXA11-AS* and NQOs in OSCC, and investigated their potential as therapeutic targets.

## 2. Results

### 2.1. Expression of HOXA11-AS and NQOs in Human OSCCs

**Figure 1:**
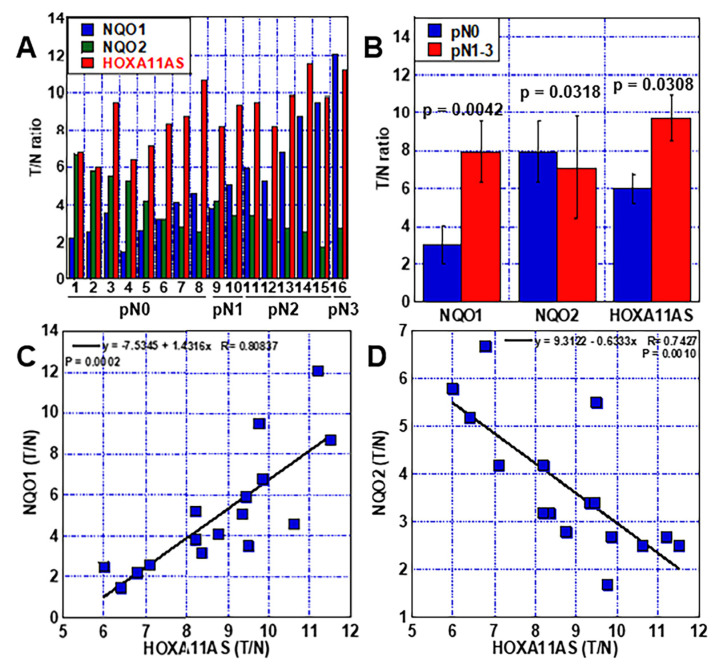
Relationship between *HOXA11-AS* and *NQO1* or *NOQ2* in 16 OSCC cases. (**A**) Relative mRNA expression of *NQO1*, *NQO2*, and *HOXA11-AS*. (**B**) Relationship between nodal metastasis and expression (T/N ratio) of *NQO1*, *NQO2*, and *HOXA11-AS*. Statistical differences were calculated by unpaired *t*-test with Welch correction. (**C**) Relationship of expression (T/N ratio) between *HOXA11-AS* and *NQO1*. (**D**) Relationship of expression (T/N ratio) between *HOXA11-AS* and *NQO2*. Regression analysis was performed using Pearson’s r-test. Error bars indicate the SD calculated using Student’s *t*-test from three independent trials. OSCC, oral squamous cell carcinoma; T/N ratio, tumor-to-normal ratio; *NQO1*, NAD(P)H: quinone oxidoreductase 1; *NQO2*, NRH: quinone oxidoreductase 2; pN0, no nodal metastasis; pN1, metastatic node <3 cm; pN2, metastatic node 3–6 cm; pN3, metastatic node >6 cm or >3 cm with extranodal invasion [13].

We first examined the expression of *HOXA11-AS* and NQOs in tissues from 16 OSCCs (Figure 1). *HOXA11-AS* expression correlated with the degree of lymph node metastasis, as did *NQO1* expression, whereas *NQO2* levels showed an inverse correlation (Figure 1A). Comparing the expression of NQO1, NQO2, and HOXA11-AS in lymph node metastasis negative cases (pN0) and positive cases (pN1-3), all showed significant differences (Figure 1B). Regression analysis results showed that there was a direct correlation between *NQO1* and *HOXA11-AS* expression, whereas *NQO2* exhibited an inverse correlation with *HOXA11-AS* expression (Figure 1C,D).

### 2.2. Role of HOXA11-AS in NQO1 Activation in Human OSCC Cell Lines

**Figure 2:**
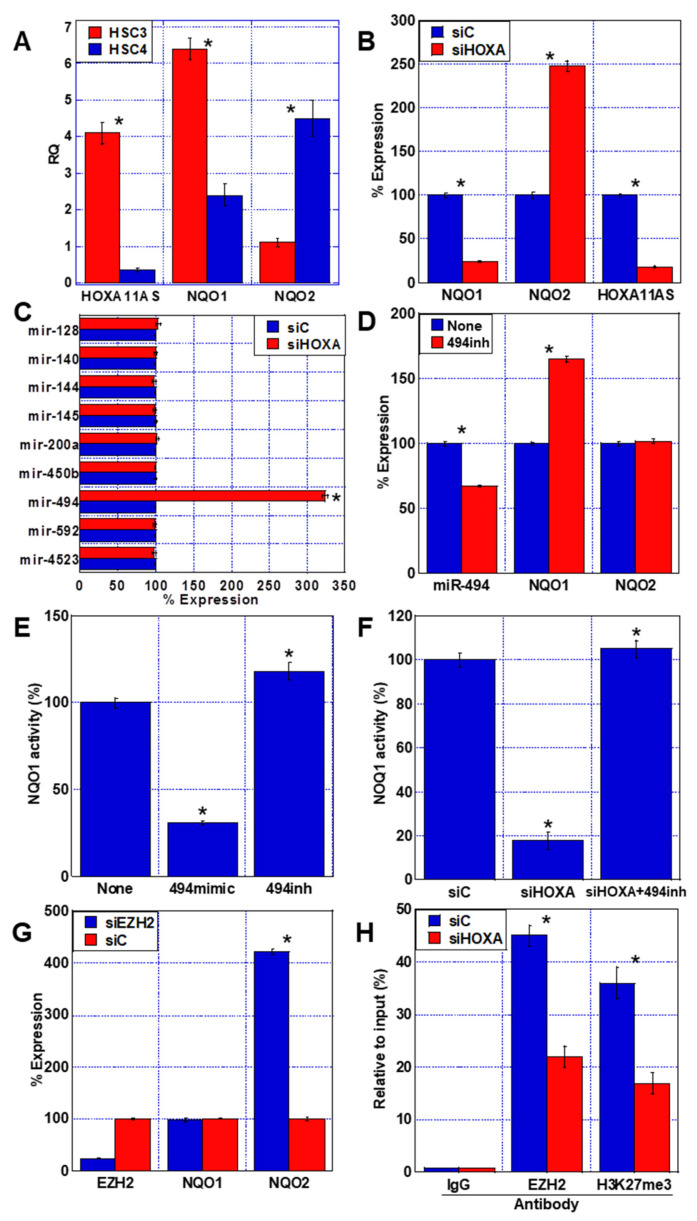
Roles of *HOXA11-AS* in expression of *NQO1* and *NQO2*. (**A**) Expression of *HOXA11-AS*, *NQO1*, and *NQO2* RNAs in HSC3 and HSC4 human OSCC carcinoma cell lines. (**B**) Effect of knockdown of *HOXA11-AS* on expression of *HOXA11-AS*, *NQO1*, and *NQO2* RNAs in HSC3 cells. (**C**) Effect of knockdown of *HOXA11-AS* on expression of various microRNAs associated with *NOQ1* expression in HSC3 cells. The references are shown in Table 1. (**D**) Effect of inhibiting *miR-494* on the expression of *miR-494*, *NQO1*, and *NQO2* in HSC3 cells. (**E**) Effect of *miR-494* mimic and *miR-494* inhibitor in HSC3 cells. (**F**) Effect of *HOXA11-AS* knockdown and *miR-494* inhibition on NQO1 activity. (**G**) Effect of *EZH2* knockdown on expression of *EZH2*, *NQO1*, and *NQO2* in HSC3 cells. (**H**) Chromatin immunoprecipitation of *NQO2* in *HOXA11-AS* knockdown HSC3 cells. Error bars indicate the SD calculated using Student’s *t*-test from three independent trials. Asterisk, *p* < 0.05. OSCC, oral squamous cell carcinoma; *NQO1*, NAD(P)H: quinone oxidoreductase 1; *NQO2*, NRH: quinone oxidoreductase 2; RQ, relative quantity; siHOXA, small interfering RNA for *HOXA11-AS*; siC, control small interference RNA; 494 mimic, miR-494 mimic; 494 inh, miR-494 inhibitor; *EZH2*, enhancer of zeste homolog 2; siEZH2, short interference RNA for *EZH2*; H3K27me3, trimethylation of histone H3 at lysine 4.

Next, we determined the expression of *HOXA11-AS* and the NQOs in the human OSCC cell lines HSC3 (highly metastatic) and HSC4 (poorly metastatic). We observed higher expression of *HOXA11-AS* and *NQO1*, but lower expression of *NQO2*, in HSC3 compared to HSC4 cells (Figure 2A). When *HOXA11-AS* was knocked down in HSC3 cells using siRNA, *NQO1* expression decreased to 25% of that in the siControl (siC) cells, but *NQO2* expression increased by 2.5-fold (Figure 2B).

We speculated that the ability of *HOXA11-AS* to upregulate *NQO1* might be due to its role as a microRNA sponge and we therefore examined the effect of knocking down *HOXA11-AS* in HSC3 cells on the expression of nine types of miRNAs that have previously been reported to upregulate *NQO1* [14,15,16,17,18,19,20,21,22]. Our results showed that only *miR-494* expression was enhanced by *HOXA11-AS* knockdown (Figure 2C). Knocking down *miR-494* in HSC3 cells increased *NQO1* expression but did not alter *NQO2* expression (Figure 2D). When we examined the effect of *miR-494* on NQO1 activity, we observed that the miR-494 mimic decreased NQO1 activity, whereas the miR-494 inhibitor increased its activity (Figure 2E). Furthermore, NQO1 activity was decreased by *HOXA11-AS* knockdown alone but was enhanced when *HOXA11-AS* knockdown was combined with the *miR-494* inhibitor (Figure 2F).

### 2.3. Role of HOXA11-AS in NQO2 Repression in Human OSCC Cell Line

We next considered that the suppression of *NQO2* expression by *HOXA11-AS* may be due to an EZH2-mediated epigenetic mechanism and examined the effect of *EZH2* knockdown in HSC3 cells (Figure 2G). *EZH2* knockdown promoted *NQO2* expression but did not alter *NQO1* expression. Moreover, the binding of EZH2 and trimethylated H3K27 to the *NQO2* promoter DNA was reduced by *HOXA11-AS* knockdown (Figure 2F).

Together, our analyses indicate that *HOXA11-AS* sponging of *miR-494* upregulates *NQO1* expression, whereas *NQO2* expression is suppressed by *HOXA11-AS*-induced EZH2 inhibition of the *NQO2* gene promoter and H3K27 trimethylation.

### 2.4. Effect of HOXA11-AS on Malignant Phenotypes of OSCC Cells

**Figure 3:**
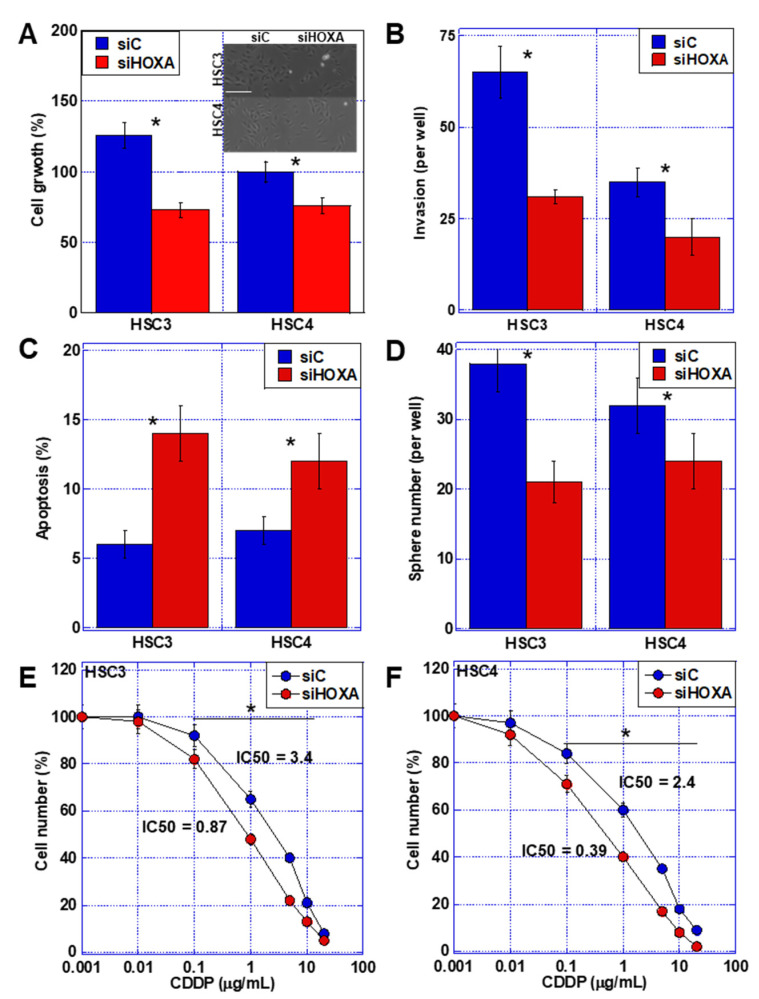
Effect of *HOXA11-AS* knockdown on malignant phenotypes in OSCC cells. (**A**–**D**) Effect of *HOXA11-AS* knockdown on cell growth (**A**), invasion (**B**), apoptosis (**C**), and sphere formation (**D**). (Inset in (**A**)) Cell morphology. Scale bar, 50 μm. (**E**,**F**) Effect of *HOXA11-AS* knockdown on sensitivity to CDDP in HSC3 (**E**) and HSC4 (**D**) cells. Error bars indicate the SD calculated using Student’s *t*-test from three independent trials. Asterisk, *p* < 0.05. OSCC, oral squamous cell carcinoma; siHOXA, small interfering RNA for *HOXA11-AS*; siC, control small interfering RNA; CDDP, cisplatin.

To examine the effects of *HOXA11-AS* on the malignant phenotype of OSCC cells, we knocked down *HOXA11-AS* in HSC3 and HSC4 cells (Figure 3). *HOXA11-AS* knockdown reduced cell proliferation and invasive capacity to a more pronounced extent in HSC3 cells (Figure 3A,B). Apoptosis was increased in both cell types (Figure 3C), while sphere-forming ability was decreased by knockdown of *HOXA11-AS* (Figure 3D), and the changes in both assays occurred to a greater degree in HSC3 compared to HSC4 cells. Furthermore, when sensitivity to CDDP was examined, sensitivity was enhanced in both cells (Figure 3E,F).

### 2.5. Role of NQO1 in OSCC Cell Lines

**Figure 4:**
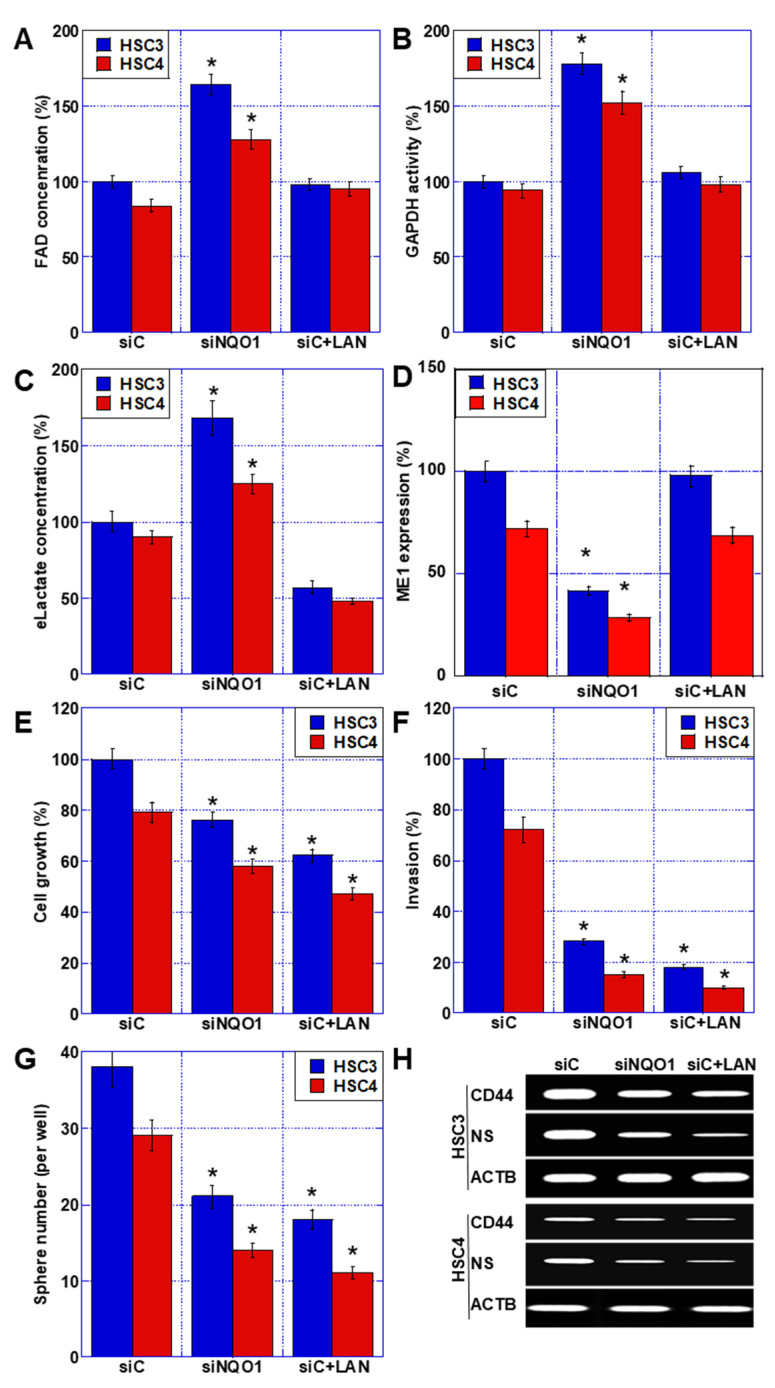
Role of *NQO1* in malignant phenotypes in OSCC cells. (**A**–**D**) Effect of *NQO1* knockdown or ME1 inhibition (LAN, 1 μM) for 48 h on FAD concentration (**A**), GAPDH activity (**B**), extracellular lactate (**C**), and ME1 expression (**D**). (**E**–**G**) Effect of *NQO1* knockdown or ME1 inhibition on cell growth (**E**), invasion (**F**), and sphere formation (**G**). (**H**) Effect of *NQO1* knockdown or ME1 inhibition on expression of CD44 and NS. Error bars indicate the SD calculated using Student’s *t*-test from three independent trials. Asterisk, *p* < 0.05. OSCC, oral squamous cell carcinoma; *NQO1*, NAD(P)H: quinone oxidoreductase 1; siNQO1, small interfering RNA for *NQO1*; siC, control small interfering RNA; LAN, lanthanide; ME, malic enzyme; FAD, flavin adenine dinucleotide; GAPDH, glyceraldehyde-3-phosphate dehydrogenase; eLactate, extracellular lactate; NS, nucleostemin.

NQO1 requires FAD in enzymatic reactions. FAD concentration in the cytoplasm increased with NQO1 knockdown (Figure 4A). In contrast, NQO1 knockdown increased the activity of GAPDH, a cytosolic glycolytic enzyme, which requires FAD for its reaction (Figure 4B). Extracellular lactate, which indicates glycolytic activity, increased (Figure 4C). The expression of malic enzyme (ME)-1, an enzyme involved in glutaminolysis (an alternate lactate fermentation pathway), was reduced by 38% following *NQO1* knockdown (Figure 4D). It has been suggested that NQO1 activity inhibits glycolysis and enhances glutaminolysis through the promotion of ME1 expression. When HSC3 and HSC4 cells were treated with ME1-inhibitory lanthanides [23], there was no change in FAD concentration and GAPDH activity, but extracellular lactate concentration decreased alongside ME1 repression (Figure 4A–D).

We also examined the roles of NQO1 and ME1 in mediating the malignant phenotypes promoted by *HOXA11-AS* (Figure 4E–H). Cell proliferation, invasive ability, apoptotic viability, sphere-forming ability, and expression of the stem cell markers CD44 and NS were all suppressed by both *NQO1* knockdown and ME1 inhibition. The degree of suppression was more pronounced in the presence of ME1 inhibition. Together, these results suggest that the promotion of malignant phenotypes by the *HOXA11-AS*–NQO1 axis is influenced by glutaminolysis.

### 2.6. Role of NQO2 in OSCC Cell Lines

**Figure 5:**
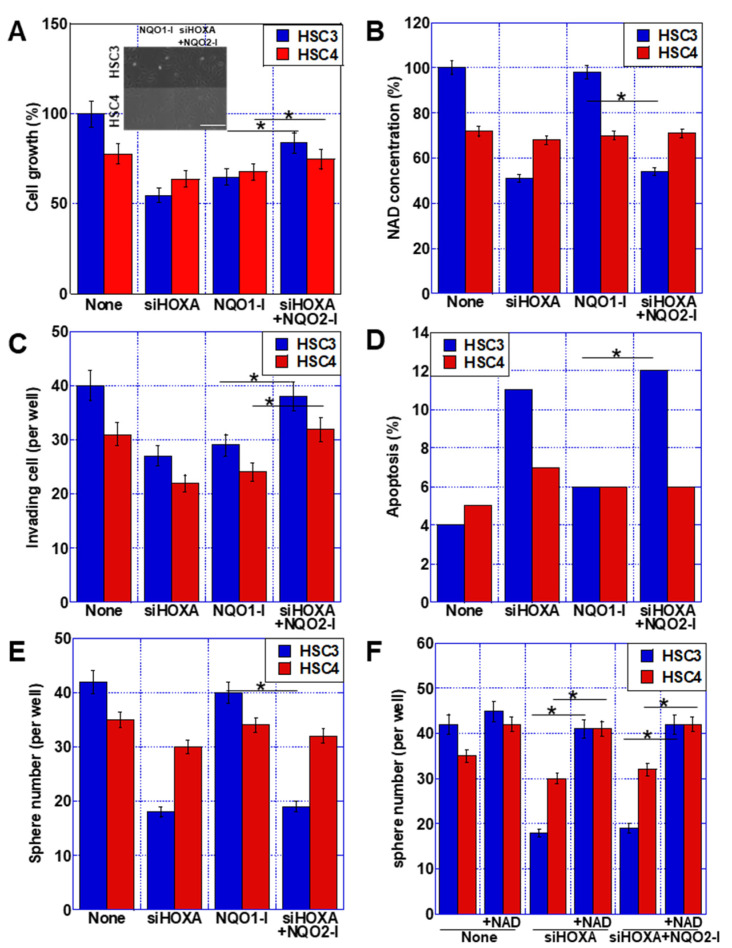
Differential roles of NQO1 and NQO2 in OSCC cells. To compare the roles of NQO1 and NQO2 in malignant phenotypes, cells were treated with siHOXA, NQO1-I (1 nM), or siHOXA+NQO2-I (15 nM) for 48 h. (**A**–**E**) Effects of these treatments on cell growth (**A**), NAD concentration (**B**), invasion (**C**), apoptosis (**D**), and sphere formation (**E**). (Inset in (**A**)) Cell morphology. Scale bar, 50 μm. (**F**) The effect of NAD on sphere formation. Error bars indicate the SD calculated using Student’s *t*-test from three independent trials. Asterisk, *p* < 0.05. OSCC, oral squamous cell carcinoma; *NQO1*, NAD(P)H: quinone oxidoreductase 1; *NQO2*, NRH: quinone oxidoreductase 2; siHOXA, small interfering RNA for *HOXA11-AS*; NQO1-I, NQO1 inhibitor (dicumarol); NQO2-I, NQO2 inhibitor (S29434); NAD, nicotinamide adenine dinucleotide.

To examine the action of NQO2, we treated the OSCC cells with *HOXA11-AS* siRNA, an NQO1 inhibitor (dicumarol), or an NQO2 inhibitor (S29434) alongside *HOXA11-AS* siRNA (to inhibit NQO2 activity upregulated by *HOXA11-AS* knockdown) (Figure 5). Our analyses show that *HOXA11-AS* knockdown and NQO1 inhibition resulted in a similar suppression of cell growth, but *HOXA11-AS* knockdown + NQO2 inhibition resulted in only mild growth suppression (Figure 5A). Intracellular NAD concentration decreased in response to *HOXA11-AS* knockdown and *HOXA11-AS* knockdown + NQO2 inhibition, but there was very little change in the presence of the NQO1 inhibitor (Figure 5B). *HOXA11-AS* knockdown and NQO1 inhibition suppressed invasion to the same extent; however, *HOXA11-AS* knockdown + NQO2 inhibition resulted in milder suppression of invasion (Figure 5C). In contrast, apoptosis was increased by *HOXA11-AS* knockdown and *HOXA11-AS* knockdown + NQO2 inhibition, but to a lesser extent by NQO1 inhibition (Figure 5D). Furthermore, *HOXA11-AS* knockdown and *HOXA11-AS* knockdown + NQO2 inhibition reduced the sphere-forming ability of the cells, but this tumor characteristic was only slightly diminished by NQO1 inhibition (Figure 5E). The decreases in sphere-forming ability shown in Figure 5E were rescued by the addition of NAD (Figure 5F).

These findings suggest that *NQO1* overexpression is the main factor involved in *HOXA11-AS*-mediated cell proliferation and invasion, while *NQO2* suppression is involved in the enhancement of stemness characteristics. Furthermore, our data also suggest that the increased levels of NAD resulting from decreased *NQO2* expression are involved in promoting features of stemness.

### 2.7. Effect of HOXA11-AS Treatment of OSCC

**Figure 6:**
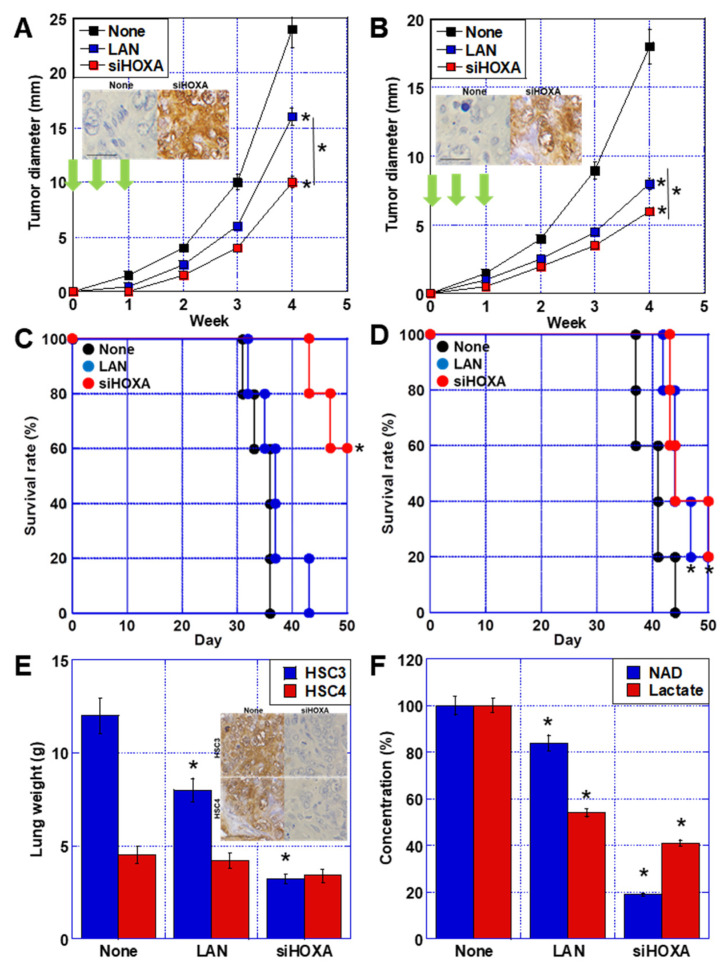
Effect of targeting *HOXA11-AS* on tumor growth and metastasis of OSCC cells. (**A**,**B**) Subcutaneous HSC3 or HSC4 tumors were treated with liposome-encapsulated siHOXA11-AS (20 pmol/mouse, i.p.) or lanthanide (0.5 μmol/kg body weight, i.p.) on Day 1, 3, and 7. (Inset) Immunostaining of NQO2. Scale bar, 50 μm. (**C**,**D**) Survival of mice in “no treatment” control (None), lanthanide, and siHOXA11-AS groups. (**E**) In the lung metastasis model, mice were treated with siHOXA11-AS or lanthanide on Day 1, 3, and 7. (Inset) Immunostaining of NQO2. Scale bar, 50 μm. (**F**) Intratumor concentration of NAD and lactate in the subcutaneous tumors. Error bars indicate SD calculated using Student’s *t*-test for five mice. Asterisk, *p* < 0.05. OSCC, oral squamous cell carcinoma; siHOXA, small interfering RNA for *HOXA11-AS*; LAN, lanthanide; NAD, nicotinamide adenine dinucleotide.

Finally, we performed experiments in a mouse model to examine the potential of *HOXA11-AS* as a therapeutic target (Figure 6). In the highly metastatic HSC3 subcutaneous tumor model, *HOXA11-AS* knockdown inhibited tumor growth by 25% more than lanthanide inhibition of ME1, a putative mediator of NQO1 (Figure 6A). In contrast, in the low metastatic HSC4 line which expresses low levels of *HOXA11-AS*, *HOXA11-AS* knockdown only increased tumor growth by 11% over lanthanides (Figure 6B). When we compared the effects of the treatments on mouse survival, we observed that lanthanide treatment only increased the mean survival time of mice harboring HSC3 tumors by 2 days compared to the control group, whereas the *HOXA11-AS* knockdown group exhibited an increase in mean survival time of 12 days over a 7-week period, with 60% of the mice still alive at the end of the experiment compared with 0% in the control and lanthanide groups (Figure 6C). In contrast, in mice carrying HSC4 tumors, both the *HOXA11-AS* knockdown and lanthanide groups displayed improved survival compared with the control group, but there was no significant difference between them (Figure 6D).

In the lung metastasis model induced by tail vein inoculation, mice with HSC4 tumors exhibited a decrease in lung weight of 7% in the lanthanide group and 22% in the *HOXA11-AS* knockdown group (Figure 6E). In contrast, mice with HSC3 metastases displayed a reduction in lung weight of 29% in the lanthanide group and 81% in the knockdown group. NAD concentrations in mice with HSC3 tumors decreased by 17% following lanthanide treatment, whereas a 79% decrease was observed with *HOXA11-AS* knockdown (Figure 6F). In addition, lactate concentration decreased by 47% and 60% in the lanthanide and knockdown groups, respectively.

Thus, mice harboring HSC3 tumors (that express high levels of *HOXA11-AS*) benefited substantially more from *HOXA11-AS* knockdown with respect to both tumor growth and metastasis than those with HSC4 tumors, which exhibit low *HOXA11-AS* expression. In addition, compared to the lanthanide-mediated suppression of NQO1 alone, the inhibition of both NQO1 and NQO2 by *HOXA11-AS* knockdown markedly enhanced the suppression of tumor growth and metastasis, with a particularly strong effect in the latter.

## 3. Discussion

In this study, we examined the role of *HOXA11-AS* as a regulator of NQOs in OSCC. Our findings show that *HOXA11-AS* had opposing effects on the NQOs, promoting *NQO1* expression and suppressing the expression of *NQO2*. The results showed that they promoted proliferation, invasion, survival of cancer cells, induction of drug resistance, and increased stemness, which in turn promoted OSCC progression and metastasis.

Our data indicate that the upregulation of *NQO1* in *HOXA11-AS*-expressing cells is mediated by the ability of the lncRNA to sponge miR-494, thus alleviating its suppression of *NQO1*. The action of “sponging”, when an lncRNA binds to a microRNA and suppresses its action, is an important regulatory feature of lncRNAs [24]. *HOXA11-AS* has been reported to act as a sponge for many microRNAs, including *miR-454-3p* (resulting in upregulation of *c-Met* expression) [25], and *miR-148b-3p* (thus upregulating *IGFBP5*) [26]. In contrast, *miR-494* is known to be sponged by several lncRNAs. Suppression of *miR-494* by the lncRNA *PCAT29* promotes *PTEN* expression [27], while a similar action by the lncRNA *SBF2-AS1* promotes *FGFR2* expression [28]. The downregulation of *NQO1* by *miR-494* has been shown to inhibit the Nrf2 signaling pathway [20], and we previously reported that *miR-494* induces a quiescent state in cancer cells by suppressing oxidative phosphorylation [29].

Studies have demonstrated that lncRNAs such as *HOXA11-AS* function as protein scaffolds for polycomb repressive complex 2, lysine-specific histone demethylase 1, and DNA methyltransferase 1, as well as recruiting EZH2 to the promoter DNA of genes, thereby regulating epigenetic gene expression [8,30]. Our results revealed that, unlike its upregulation of *NQO1*, *HOXA11-AS* recruited EZH2 to the *NQO2* gene promoter to suppress the expression of *NQO2*. Our data indicate that the regulation of *NQO2* expression involves H3K27 trimethylation of histones binding to the promoter DNA of the *NQO2* gene. H3K27me3 epigenetically represses gene expression [31]. It has been suggested that *NQO2* is regulated by epigenetic expression [32], but this study indicated that its expression is regulated by H3K3me3.

In our data, *ME1* expression was induced along with NQO1 overexpression. This has the effect of compensating for the reduction in the glycolysis caused by GAPDH activity due to FAD consumption of NQO1 by glutaminolysis. Glutaminolysis provides a substrate for lactate fermentation, and promotes anaerobic energy metabolism. Glutaminolysis is elevated in cancer cells and correlates with cancer metastasis [33]. We previously reported that in OSCC, enhanced glutaminolysis mediated by *ME1* expression leads to budding, enhanced stemness, induction of EMT, and acquisition of metastatic potential at the leading edge of tumor invasion [23,34]. The results from this study indicate that *HOXA11-AS*-mediated *NQO1* expression is one of the causes of *ME1* upregulation.

It has been reported that decreased *NQO2* expression correlates with colorectal cancer liver metastasis and lymph node metastasis [7,35]. *NQO2* knockout mice developed myelodysplastic syndrome as a result of bone marrow cell hyperplasia and decreased apoptosis [36]. NQO2 differs from NQO1 in that it requires NRH as a coenzyme [10]. NRH is a precursor of NAD [37] and increases intracellular NAD levels [38,39]. Our experiments showed that an increase in *NQO2* following *HOXA11-AS* knockdown resulted in a decrease in NAD concentration. This suggests that NQO2 activation reduces NRH levels, resulting in decreased NAD levels. NAD plays an important role as a coenzyme in energy metabolism reactions, such as glycolysis, the TCA cycle, and oxidative phosphorylation, and is also required for PARP-mediated DNA repair [40]. Studies have demonstrated that NAD is required for the survival of cancer tissues and cancer stem cells [41,42]. Our study also confirmed this, as we observed that a reduction in stemness caused by *HOXA11-AS* knockdown was restored by *NQO2* knockdown or NAD supplementation.

We examined the differential effects of these two NQOs by comparing the results of inhibiting NQO1 with dicoumarol, and NQO2 with *HOXA11-AS* knockdown. Our data showed that NQO1 promoted cell proliferation, invasive capacity, viability, and drug resistance. In contrast, NQO2 enhanced stemness. *HOXA11-AS* induces a strong malignant phenotype in OSCC cells by simultaneously upregulating *NQO1* and downregulating *NQO2*. In our therapeutic experiments using mouse models, knockdown of *HOXA11-AS* markedly suppressed tumor growth and metastasis.

We compared the results of inhibition of NQO1 with dicoumarol and NQO2 with HOXA11-AS knockdown to examine the differences in the actions of these two NQOs. The results revealed that NQO1 promotes cell proliferation, invasive capacity, viability, and drug resistance. In contrast, NQO2 promoted stemness, and HOXA11-AS induced a strong malignant phenotype in OSCC cells by simultaneously upregulating NQO1 and downregulating NQO2. In therapeutic experiments using mouse models, knockdown of HOXA11-AS markedly suppressed tumor growth and metastasis.

Our data show that HOXA11-AS upregulates NQO1 expression by sponging miR-494 and downregulates NQO2 expression by EHZ2-mediated H3K27 trimethylation; overexpression of NQO promotes malignant phenotypes by promoting glutaminolysis, and suppression of NQO2 expression enhanced cancer stemness by increasing intracellular NAD levels. Furthermore, NQO1 plays a role in neutralizing oxidative stress and detoxification, which may promote cancer cell survival [10]. NQO1 upregulation and NQO2 downregulation are provided synchronously by HOXA11-AS, resulting in the phenotype alterations described above and promoting cancer metastatic potential. The HOXA11-AS–NQO1/NQO2 axis is emphasized as a novel metastasis-promoting mechanism in OSCC.

Oncogenic lncRNAs, which are upregulated in cancer, are expected to be diagnostic markers, prognostic factors, and even novel therapeutic molecular targets [43]. HOXA11AS is one of the 16 most dysregulated lncRNAs in head and neck cancer and has attracted attention as a therapeutic target [44]. Attempts to apply lncRNA targeting to therapy have included the use of antisense oligonucleotides and small molecules to block the functional interactions between lncRNAs and proteins, and inactivation of lncRNA-encoding genes and the transcripts based on the CRISPR-Cas system are under development [45]. Therefore, we believe that *HOXA11-AS* may be a novel molecular target for the treatment of OSCC.

## 4. Materials and Methods

### 4.1. Surgical Specimens

Fresh surgical specimens frozen at −80 °C from 16 primary OSCCs treated at the Nara Medical University Hospital were randomly selected for use in this study. Tumor stage was determined using the TNM classification system [13], and the personal information of the patients was anonymized by the staff. All procedures were performed in accordance with the Ethical Guidelines for Human Genome/Gene Research enacted by the Japanese Government and were approved by the Ethics Committee of Nara Medical University (approval number 937, 20 October 2010).

### 4.2. Cell Culture

The human tongue squamous cell carcinoma cell lines HSC3 and HSC4 were purchased from the Health Science Research Resources Bank (Osaka, Japan). The cells were maintained in Dulbecco’s modified Eagle medium (DMEM) containing 450 mg/dL glucose and 10% fetal bovine serum in a 5% CO_2_ atmosphere at 37 °C.

### 4.3. Reagents

These reagents were purchased from the indicated suppliers: *miR-494* mimic and *miR-494* inhibitor (Invitrogen, Carlsbad, CA, USA); ME inhibitor, lanthanide (1 μM, WAKO, Osaka, Japan) (Nakashima C, Cancer Sci. 2018); cisplatin (CDDP, WAKO); NQO1 inhibitor (NQO1-I, dicumarol Sigma-Aldrich Chemical Co., St. Louis, MO, USA); NQO2 inhibitor (NQO2-I, S29434, Sigma); and beta-NAD (MP Biomedicals, Inc., Irvine, CA, USA).

### 4.4. Cell Proliferation, Cell Infiltration, and Apoptosis

To assess cell proliferation, cell numbers were determined using an autocytometer (CDA-1000; Sysmex, Kobe, Japan) or MTS [3-(4,5-dimethylthiazol-2-yl)-5-(3-carboxymethoxyphenyl)-2-(4-sulfophenyl)-2H-tetrazolium] assay. MTS assays were performed using the Celltiter 96 Aqueous One Solution Cell Proliferation Assay kit (Promega Biosciences, Inc., San Louis Obispo, CA, USA). Wound healing assays were performed to assess cell infiltration. The area of cell migration was measured using digitally captured images. In some experiments, the cells were treated with lanthanide for 48 h at 37 °C. Apoptosis was assessed by examining 1000 cells stained with Hoechst 33342 dye (Life Technologies, Carlsbad, CA, USA) and viewed using a fluorescent microscope.

### 4.5. Sphere Formation Assay

Cells (1000 cells/well) were seeded onto uncoated bacteriological 35 mm dishes (Corning Inc., Corning, NY, USA) in 3D Tumorsphere Medium XF (Sigma) [46]. After seven days of culture, images of the spheres were acquired using an inverted microscope coupled with a camera (Carl Zeiss, Göttingen, Germany). The captured images were analyzed using a computer, and the number of spheres was counted using ImageJ software (version 1.52; NIH, Bethesda, MD, USA).

### 4.6. Small Interfering RNA

Stealth Select RNAi (siRNA) targeting human *HOXA11-AS*, *NQO1*, and *EZH2* was purchased from Sigma. AllStars Negative Control siRNA was used as the control (Qiagen, Valencia, CA, USA). The cells were transfected with 10 nM siRNA using Lipofectamine 3000 (Thermo Fisher Scientific, Waltham, MA, USA) according to the manufacturer’s recommendations.

### 4.7. Reverse Transcription Polymerase Chain Reaction (RT-PCR)

Total RNA (1 μg) was used to synthesize cDNA using the ReverTra Ace quantitative PCR (qPCR) RT kit (Toyobo, Osaka, Japan). The PCR reaction was performed according to the manufacturer’s instructions. PCR products were electrophoresed on 2% agarose gels and visualized using ethidium bromide. The primer sets used are listed in Table 1. Primers were synthesized by Sigma-Aldrich (Ishikari, Japan).

### 4.8. qRT-PCR for lncRNA

Total cellular RNA was isolated from primary OSCC tissues using TRIzol reagent (Invitrogen) and reverse-transcribed using the Prime Script RT reagent kit together with gDNA Eraser (Perfect Real Time; Takara, Kyoto, Japan) in accordance with the manufacturer’s instructions. *HOXA11-AS* expression was analyzed using qRT-PCR, with reactions performed in triplicate using a SYBR Green PCR kit (Takara). Glyceraldehyde3-phosphate dehydrogenase (*GAPDH*) mRNA was used as the internal control. The primer sets are listed in Table 1.

### 4.9. Detection of miRNA

A mirVana™ miRNA isolation kit was used to extract miRNAs, according to the manufacturer’s protocol (Thermo Fisher Scientific). To quantify miRNA expression levels, RT-PCR was performed using the TaqMan miRNA reverse transcription kit (Applied Biosystems) and Pri-miRNA Assay kit (Hs04225959_pri, Applied Biosystems) according to the manufacturer’s protocols. The primer sets are listed in Table 1.

### 4.10. Protein Extraction

To prepare whole-cell lysates, the cells were washed twice with cold PBS and harvested. The cells were lysed with 0.1% NP-40-added RIPA buffer (Thermo Fisher) [47]. Protein assays were performed using the Protein Assay Rapid Kit (Wako).

### 4.11. Determination of Lactate, NAD, and FAD Concentrations, and Activity of NQO1 and GAPDH

Concentrations of lactate, NAD, and FAD were determined using a D-lactate assay kit (Cayman Chemical, Ann Arbor, MI, USA), NAD/NADH assay kit (Dojindo, Kumamoto, Japan), and FAD ELISA kit (As One, Tokyo, Japan), respectively.

The activities of NQO1 and GAPDH were determined using NQO1 and GAPDH activity assay kits (Abcam, Cambridge, UK), respectively. All reactions were performed according to the manufacturer’s instructions.

### 4.12. Chromatin Immunoprecipitation Assay

A chromatin immunoprecipitation assay was performed in HSC3 cells using a commercial kit (Sigma). After cross-linking with 1% formaldehyde at 37 °C for 10 min, the cells were harvested in sodium dodecyl sulfate lysis buffer and the DNA was sheared to fragments of 500 bp by sonication. Pre-cleared chromatin was incubated overnight with antibodies against EZH2, H3K27me3, or non-specific IgG. Protein G-agarose beads were then added and incubated at 4 °C for 1 h. After reversing the cross-links, the DNA was isolated and used for PCR. The specific primers used for PCR detection of the *NQO2* promoter are shown in Table 1.

### 4.13. Animal Models

Male 5-week-old BALB/c nude mice were purchased from Japan SLC (Shizuoka, Japan). Mice were maintained in accordance with the institutional guidelines approved by the Committee for Animal Experimentation of Nara Medical University and the current regulations and standards established by the Ministry of Health, Labor, and Welfare (approval number 12047, 17 July 2017).

To prepare a subcutaneous tumor model, HSC3 and HSC4 cells (1 × 10^7^ cells) suspended in Hank’s balanced salt solution (100 μL, Sigma-Aldrich) were inoculated into the scapular subcutaneous tissue of the mice. At week 4, the tumors were excised for analysis. To prepare a lung metastasis model, HSC3 and HSC4 cells (1 × 10^6^ cells) suspended in Hank’s balanced salt solution (50 μL, Sigma-Aldrich) were inoculated into the caudal vein. At week 4, the lungs were excised for analysis. Each experimental group contained five mice.

For tumor treatment, lanthanide (0.5 μmol/kg body weight in 200 μL) or *HOXA11-AS* siRNA (Qiagen, 20 pmol encapsulated with liposome, Nippon-Oil&Fats Co., Tokyo, Japan) was injected intraperitoneally on days 1, 3, and 7 [48].

### 4.14. Statistical Analysis

Statistical significance was assessed using Student’s *t*-test with the assumption of a Gaussian distribution according to the Kolmogorov and Smirnov method or unpaired *t*-test with Welch correction. Regression analysis was performed using Pearson’s r analysis with the assumption of a Gaussian distribution. Analyses were performed using the InStat software (GraphPad, Los Angeles, CA, USA). Survival curves were calculated using the Kaplan–Meier model (StatView 4.5, Abacus Concepts, Inc., Berkeley, CA, USA). Differences in survival times were calculated using the Cox proportional hazards model (StatView 4.5). Statistical significance was defined as *p* < 0.05.

## Figures and Tables

**Table 1:** Primer sets.

Gene Name	Gene ID		Forward (5′–3′)	Reference
Promoter DNA				
NQO2 promoter	AY334547.1	Upper	TGGCATCTCACAAAGGACAG	
		Lower	GCCGCTGGTGTACTGGTATT	
RNA				
*nucleostemin (NS)*	BC001024.2	Upper	ATTGCCAACAGTGGTGTTCA	
		Lower	AATGGCTTTGCTGCAAGTTT	
*CD44*	FJ216964.1	Upper	CATTCAAATCCGGAAGTGCT	
		Lower	GTTGCCAAACCACTGTTCCT	
*NQO1*	BC007659.2	Upper	AAAGGACCCTTCCGGAGTAA	
		Lower	CCATCCTTCCAGGATTTGAA	
*NQO2*	BC006096.2	Upper	CACACCAGGAACCCAAGTCT	
		Lower	TTGTAGGCTTCGTGGGTTTC	
*ACTB*	NM_001101.3	Upper	GGACTTCGAGCAAGAGATGG	
		Lower	AGCACTGTGTTGGCGTACAG	
lncRNA				
*HOXA11AS*	NR_002795.2	Upper	TCTCCTGGAGTCTCGCATTT	
		Lower	TCGGAAGTGACCATGAATGA	
miRNA				
*mir-128*	NR_029672.1	Upper	GCCGTAGCACTGTCTGAGAG	[14]
		Lower	GCAGCTGAAAAAGAGACCGG	
*mir-140*	NR_029681.1	Upper	TGTGTCCTGCCAGTGGTTTT	[15]
		Lower	GTCCGTGGTTCTACCCTGTG	
*mir-144*	NR_029685.1	Upper	AGTTTGCGATGAGACACTACAGT	[16]
		Lower	GGTGCCCGGACTAGTACATC	
*mir-145*	NR_029686.1	Upper	CTTGTCCTCACGGTCCAGTT	[17]
		Lower	TTCCTGGGAAAACTGGACCG	
*mir-200a*	NR_029834.1	Upper	AGCATCTTACCGGACAGTGC	[18]
		Lower	TGGGAAATCCAGCACTGTCC	
*mir-450b*	LM609945.1	Upper	AAGTGTATTGGGATCATTTTGCA	[19]
		Lower	ACTATGGATGCAAAATGATCCCA	
*mir-494*	NR_030174.1	Upper	CTCGAAGGAGAGGTTGTCCG	[20]
		Lower	AGAAGACAACACGGACAACCT	
*mir-592*	NR_030323.1	Upper	GCGATGATGTGTTGTGATGGC	[21]
		Lower	CGTCATGATGTTGCGTCACC	
*mir-4523*	NR_039749.1	Upper	TCGGCTGTGTGAGGACTAGA	[22]
		Lower	CTCGGCCGCCTCTAGTCC	

## Data Availability

Not applicable.

## Abbreviation

lncRNA	Long non-coding RNA
HOX	homeobox
HOXA11-AS	HOX-A11 antisense
OSCC	oral squamous cell carcinoma
*NQO1*	NAD(P)H: quinone oxidoreductase 1
*NQO2*	NRH: quinone oxidoreductase 2
*EZH2*	enhancer of zeste homolog 2
H3K27me3	trimethylation of histone H3 at lysine 4
CDDP	cisplatin
LAN	lanthanide
ME	malic enzyme
FAD	flavin adenine dinucleotide
GAPDH	glyceraldehyde-3-phosphate dehydrogenase
NAD	nicotinamide adenine dinucleotide
